# No Effect of Ambient Odor on the Affective Appraisal of a Desktop Virtual Environment with Signs of Disorder

**DOI:** 10.1371/journal.pone.0078721

**Published:** 2013-11-08

**Authors:** Alexander Toet, Martin van Schaik, Nicolet C. M. Theunissen

**Affiliations:** 1 Perceptual and Cognitive Systems, TNO, Soesterberg, The Netherlands; 2 Department of Information and Computing Sciences, University of Utrecht, Utrecht, The Netherlands; 3 Training & Performance, TNO, Soesterberg, The Netherlands; Technical University of Dresden Medical School, Germany

## Abstract

**Background:**

Desktop virtual environments (VEs) are increasingly deployed to study the effects of environmental qualities and interventions on human behavior and safety related concerns in built environments. For these applications it is essential that users appraise the affective qualities of the VE similar to those of its real world counterpart. Previous studies have shown that factors like simulated lighting, sound and dynamic elements all contribute to the affective appraisal of a desktop VE. Since ambient odor is known to affect the affective appraisal of real environments, and has been shown to increase the sense of presence in immersive VEs, it may also be an effective tool to tune the affective appraisal of desktop VEs. This study investigated if exposure to ambient odor can modulate the affective appraisal of a desktop VE with signs of public disorder.

**Method:**

Participants explored a desktop VE representing a suburban neighborhood with signs of public disorder (neglect, vandalism and crime), while being exposed to either room air or subliminal levels of unpleasant (tar) or pleasant (cut grass) ambient odor. Whenever they encountered signs of disorder they reported their safety related concerns and associated affective feelings.

**Results:**

Signs of crime in the desktop VE were associated with negative affective feelings and concerns for personal safety and personal property. However, there was no significant difference between reported safety related concerns and affective connotations in the control (no-odor) and in each of the two ambient odor conditions.

**Conclusion:**

Ambient odor did not affect safety related concerns and affective connotations associated with signs of disorder in the desktop VE. Thus, semantic congruency between ambient odor and a desktop VE may not be sufficient to influence its affective appraisal, and a more realistic simulation in which simulated objects appear to emit scents may be required to achieve this goal.

## Introduction

Desktop VEs (virtual environments) are increasingly deployed to study future design plans and the possible effects of environmental qualities and interventions on human behavior and feelings of safety in built environments [1]–[4]. Their effectiveness for these applications depends critically on their ability to correctly address the user’s affective appraisal of the represented environment. It is essential that users appraise the affective qualities of the desktop VE similar to those of its real world counterpart. Previous studies have shown that factors like simulated lighting, sound and dynamic elements have a similar influence on the affective appraisal of desktop VEs and real environments [5], [6]. Ambient scent is another important environmental characteristic that is currently lacking in most VEs. It has been shown that ambient odor can increase the sense of presence in immersive VEs [7]–[9]. Ambient scent is known to significantly affect our appraisal of real environments [10], and people have strong expectations about the way an environment should smell [11]. Hence, semantically congruent ambient odors may also be an effective tool to tune the affective appraisal of less immersive desktop VEs (e.g., by evoking implicit associations).

This study investigated if (pleasant and unpleasant) ambient odors can influence the affective appraisal of a desktop VE displaying signs of social disorder. Previous studies found that although signs of public disorder influenced the affective appraisal of audio-visual desktop VEs and their real-world counterparts in a similar way, the extent of the effect could differ significantly [4], [12], [13]. This finding seriously degrades the ecological validity of desktop VEs for this type of studies. In this study participants explored and appraised a desktop VE in conditions with either no ambient scent or with a pleasant or unpleasant semantically congruent ambient scent, to test if the presence of an ambient odor can modulate the affective appraisal of the desktop VE.

### Virtual Environments in Environmental Research

Traditional methods used in environmental psychology to assess the impact of the affective qualities of built environments on the appraisal of the environment and safety related concerns typically use surveys and interviews [14], [15], sometimes in combination with site visits [16], [17]. One of the typical questions is “*How would you feel being out here alone at night*?” Experiments in reality are often difficult to organize and perform, time consuming and costly, and may involve practical dangers and risks for the participants. Moreover, real environments are constantly changing and it is not possible to control all experimental variables (e.g., noise level, temperature, light intensity, social presence, wind, rain, etc.) and interfering factors. But most importantly, it is generally impractical, too costly or simply impossible to investigate the effects of different interventions and new environmental designs and layouts.

The limitations and safety issues associated with experiments on the affective appraisal of built environments in reality can to a large extent be avoided by the use of drawings, photographs and slides, and interactive panoramas that can help participants to mentally place themselves in the real situation [18]. Photographs and slides have for instance been used to assess the impact of urban [19]–[21] and natural [22] layout, darkness [21], [23], [24] and graffiti [25] on concerns for personal safety. Although the appraisal of photographs correlate with on-site evaluations [26], they are highly viewpoint dependent [27]. In addition, photographs fail to capture the dynamics of a real environment and its context [25]. Desktop VEs do not have these drawbacks, and offer cost-effective, safe, controlled and flexible environments that allow to investigate human response to a wide range of environmental factors without the constraints, distractions and dangers of the real world (e.g., [28]). As a result, desktop VEs are increasingly deployed to study the effects of new designs and interventions in environmental qualities and measures like CPTED (“Crime Prevention Through Environmental Design”) on human behavior and concerns for personal safety in built environments [1], [29]–[32]. A recent series of studies used desktop VEs to investigate the effects of environmental cues signaling risk of victimization on concerns for personal safety [2], [3], [33]–[36]. One of these studies addressed the ecological validity of a desktop VE for this type of research [3]. It was found that the effects of cues like graffiti, murals, and boarded-up broken windows on concerns for personal safety of local residents were similar in reality and in a virtual model of the area. Other studies that compared human response to environmental interventions in both desktop simulations and their real world counterparts found that simulated lighting levels [5], [37], and path choices [38] evoke similar responses as their real-world equivalents. Thus, it appears that the physical and affective qualities of desktop VEs influence human response in many respects in a similar way as their real-world equivalents.

Although VEs can relatively easy be implemented on immersive platforms like a CAVE [39], head mounted displays (HMD’s) and projection domes, most visualizations for urban planning and design are still displayed on desktop systems [40]. Desktop displays are relatively cheap, widely available and easy to use, while most users are familiar with these displays and their interaction devices. Desktop VEs are also preferred for communication of design plans because they can be made accessible to a large numbers of users in internet applications [41]. Although desktop VEs generally less effectively induce a sense of presence than more immersive displays [42] and seldom achieve full ecological validity [5], this need not be a significant limitation for the study of the effects of physical or social interventions in a built environment on human behavior and safety related concerns. For most purposes it suffices if the affective appraisal of the VE (or the mental imagery it evokes: [43]) is similar to that of its real-world equivalent [13], [44]. In this context the term ‘*affective appraisal*’ refers to the assessment of the environmental qualities that have the *capacity* to alter an individuals’ affective state (the *affective qualities* of the environment). Thus, affective appraisals are attributed affective qualities about possible emotional reactions evoked by the environment [18], [45], that are not necessarily accompanied by an actual affective responses [13], [46]. Since fear is based on affective appraisals (a situation must be appraised as threatening or dangerous before it will lead to fearful reactions; it is logically impossible to be afraid but not judge a situation as threatening: [47]) a VE that is affectively appraised similar to its real world equivalent may be a valid predictor of human emotions (e.g. feelings of fear).

Previous studies comparing the influence of signs of public disorder on the affective appraisal of the environment found similar effects for desktop VEs and their real-world counterparts [4], [12], [13]: both in VE and in reality, signs of disorder typically compromise feelings of safety (see also [35]). However, for desktop VEs, the effects were significantly less than expected when the participants merely regarded these details as interesting distractions [12], [13], while they were much stronger than expected when the participants over-focused on these details [4]. These effects seriously degrade the ecological validity of desktop VEs for the study of effects of environmental qualities and interventions on human behavior and safety related concerns. Possible reasons for these effects may be that in the real world the impact of signs of public disorder is typically modulated by various environmental factors and social presence [48]. For instance, their negative appraisal can be ameliorated by auditory (music), tactile (wind), and olfactory (pleasant ambient smells of fresh air and vegetation) cues, or enhanced by noise and unpleasant (e.g., garbage and urine) smells. Hence, further knowledge of and control over these sensory factors is required to enable the design and construction of desktop VEs that can effectively be tuned to elicit an affective appraisal of the represented environment that agrees with the purpose of the simulation, and that is similar to the appraisal the user would have of the corresponding situation in reality. Previous studies investigated how simulated lighting [49]–[51], sound [52] and dynamic elements [6] may contribute to this goal. This study investigates if ambient odor influences a user’s affective appraisal of a desktop VE.

### Olfaction, Affect and Attention

Odors can trigger a wide range of affective responses [53]–[55] and memories [56], and influence human judgments and behavior even when they are not consciously perceived [57]–[61]. This probably reflects the high degree of overlap between the brain structures involved in olfactory and emotional processing [62], [63]. The nature of the response is inextricably linked to odor hedonic tone (pleasantness: [64]). Pleasant odors tend to induce positive affect, whereas unpleasant odors tend to induce negative affect [65]–[67].

Odors can also modulate the affective quality of images [68]–[71]. Foul odors reduce the liking for picture and photographs [70], and this effect is strongest for pleasant and neutral images [68]. There is accumulating evidence that the brain pre-attentively makes an initial prediction of the affective ‘gist’ of a scene which in turn affects its appraisal in a top-down fashion [72]. As a result subliminally perceived odor may influence affective responses in a direct way not mediated by mood or arousal [73]. Smells can also affect the appraisal of environments, places and persons [10], [11], [74], [75], either directly through association with its contents [73], [76], [77] or indirectly through misattribution of induced affect [78]–[83]. As a result, pleasant ambient scents even tend to improve the evaluation of objects that are generally judged less favorably [73], [76].

Ambient odors (even at subthreshold levels) modulate visual attention [73], [84], [85]. Recent studies have shown that odors bias visual selective attention for *semantically congruent* visual objects [86]–[88]. It appears that crossmodal odor-object associations are activated automatically without the need for explicit odor identification [86]. Ambient odors also bias visual attention to favor stimuli that are *affectively congruent* to their hedonic quality (a case of affect-biased attention: [89]). Pleasant odors facilitate the processing of positive visual cues [90], while unpleasant odors facilitate the processing of negative cues [91] and inhibit the processing of positive cues [90]. The pre-attentive affective bias induced by ambient unpleasant odors probably serves the ecological purpose of facilitating threat detection [92].

### Olfaction and Virtual Environments

Despite the major role of scent in everyday life and its effects on our environmental appraisal, olfaction is rarely applied in the scope of VEs [93]. Early problems associated with dispersing and controlling the odorants in the environment have been resolved by recent technological developments [94]–[98]; for a review see [99] and for a current overview of available display systems see [100]. With the technological barriers to effective presentation of olfactory information overcome, VE researchers and developers now have the ability to utilize scent to create more authentic environments and scenarios [88]. Enhancing virtual environments with olfactory stimuli may create a more complex and richer user experience by heightening the sense of reality [101], [102]. It has indeed been shown that the addition of olfactory cues to an immersive VE can increase the user’s sense of presence, memory and perceived realism of the simulated environment [7]–[9]. Recent applications of olfactory enhanced VEs include high-end games and entertainment [103], [104] and clinical scenarios for (drug, alcohol, nicotine) cue reactivity assessment [105], [106] and PTSD treatment [107]. However, it is still unknown if ambient scents can influence the affective appraisal of a desktop VE [101].

### Current Study

Public disorder reflects erosion of social control and inspires fear of crime [48], [108]–[114]. Public disorder encompasses both physical and social disorder. Physical disorder includes items like dilapidated housing, vandalism, litter and vacant lots, while social disorder includes phenomena like loitering youths, rowdy behavior, public drunkenness, drug sales and prostitution. Following Park et al. [36] we conceptualize *Fear of Crime* in this study as a cue-focussed affective appraisal of victimization risk rather than an actual feeling of fear(see also [48], [114]). Hence, *Fear of Crime* is conceptualized here as the capability of a given situation to evoke safety related concerns, measured through the affective appraisal of cues in the environment that signify a potential threat [15].

The current study was performed to test if exposure to (pleasant or unpleasant) ambient odor can modulate the affective appraisal of a VE showing signs of disorder. Participants performed a walking tour through a VE while being exposed to either room air (control group), unpleasant (tar) or pleasant (cut grass) odor. Whenever they noticed signs of disorder during their walk they reported their momentary safety related concerns and their associated affective feelings. The pleasant scent (cut grass) had congruent visual and auditory representations in the simulation, since the VE showed abundant greenery and contained the occasional sound of grass mowers in the associated soundtrack. The unpleasant scent (tar) had no obvious (visual or auditory) counterparts in the simulation, but could be associated with derelict areas in general and with the occasional sounds of construction activities (hammering, sawing) in the soundtrack of the VE in particular. Since people tend to respond to an environment as a whole (a ‘molar’ environment) rather than to its individual features [13], [115]–[117], and since affective qualities are prioritized in this categorization process [117], the presence of an ambient scent with an affective (pleasant or unpleasant) loading was expected to bias the affective appraisal of the VE. More specifically, it was hypothesized that participants in the unpleasant odor condition (H1) would report more concern for crime than participants in the control condition, because unpleasant odors bias visual attention to - affectively congruent - negative cues, and (H2) they would associate more negative affect with these signs because they would –unconsciously - associate the unpleasant odor with the affective quality of the VE. In contrast, it was expected that participants in the pleasant odor condition (H3) would report less concern for crime than participants in the control condition, because the smell of cut grass would bias their attention to the – semantically congruent - greenery and thereby distract them from the signs of disorder, and (H4) they would associate less negative affect with these signs (since pleasant scents tend to improve the evaluation of objects that are otherwise judged less favorably and to improve mood).

## Methods

### Virtual Environment

**Figure 1:**
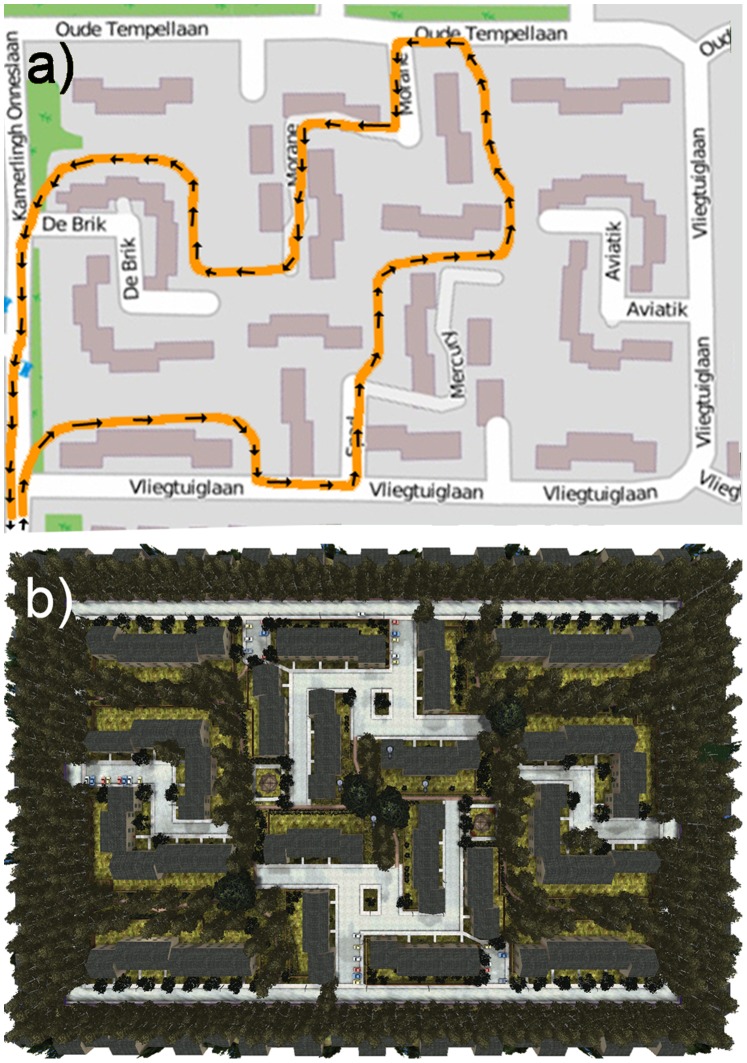
Map of the modeled area of the town of Soesterberg (www.openstreetmap.org). (a) Walking route (orange line) and walking direction (small black arrows). (b) Corresponding top-view of the virtual environment.

**Figure 2:**
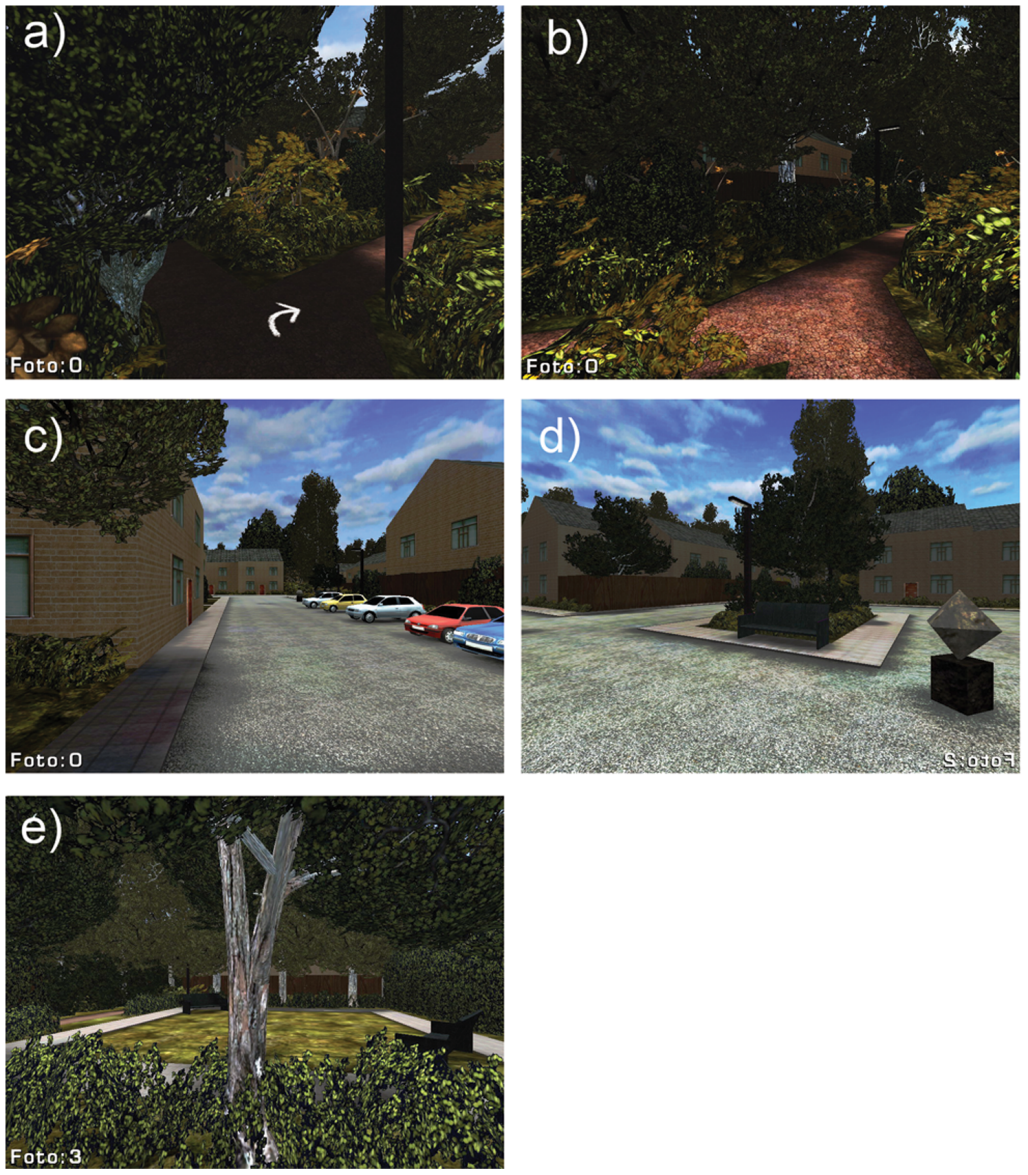
Characteristic scenes from virtual environment. (a,b) Pathways between fences enclosing the back gardens of the houses in the neighborhood. Notice the arrows marked on the ground to indicate the walking route. (c) Entrance to a small square with parking. (d) A square with benches and artwork, surrounded by houses. (e) A playground.

**Figure 3:**
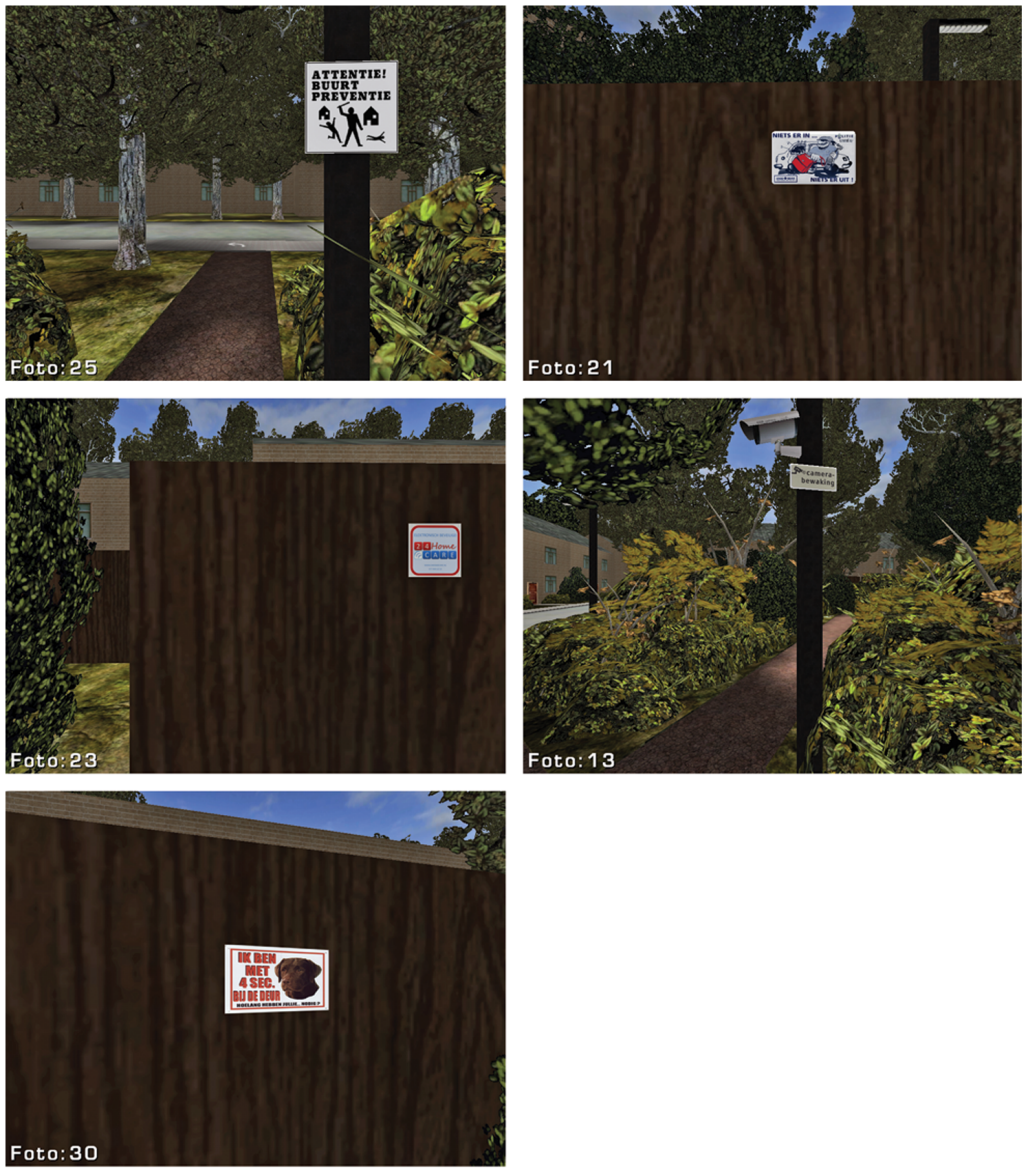
Warning signs and cameras.

**Figure 4:**
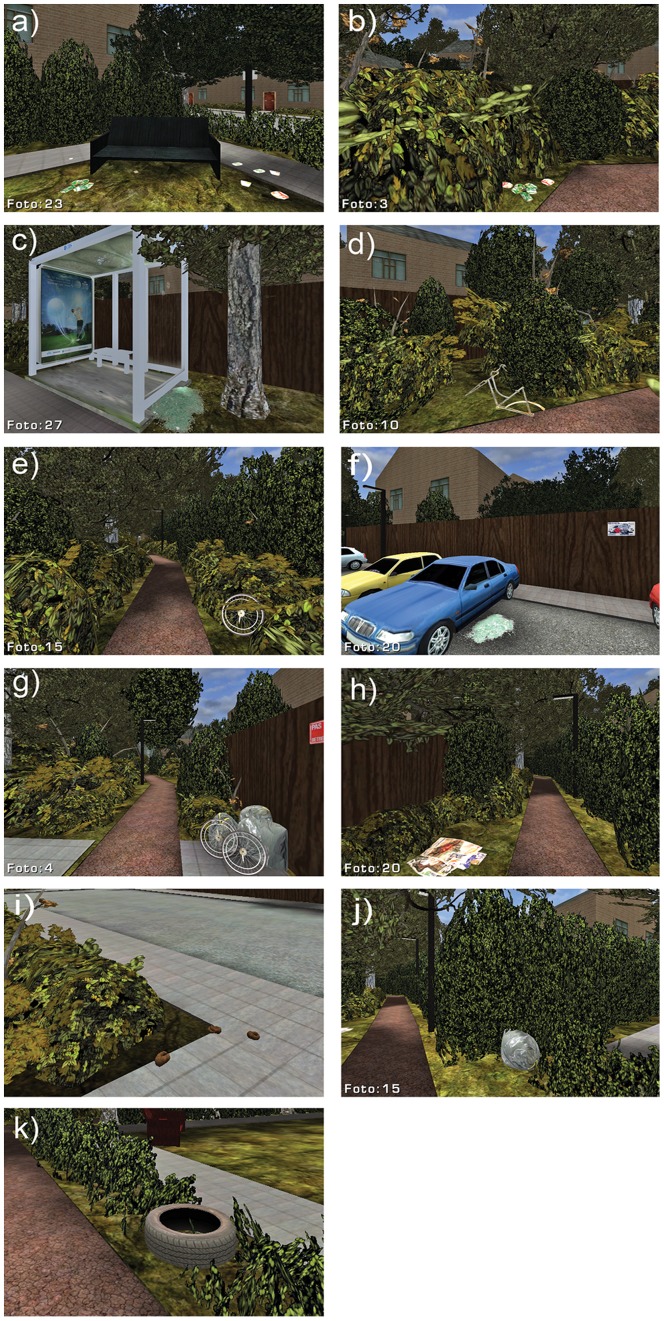
Physical incivilities in the virtual environment. Examples shown represent (a,b) empty beer cans, (c) broken glass, (d,e) bicycle parts, (f) broken car window glass, (g) garbage bags and bicycle parts, (h) paper litter, (i) dog droppings, (j) garbage bag, (k) car tire.

A small area in the town of Soesterberg, The Netherlands (with a rectangular shape and a total extent of about 200×200 m^2^; coordinates 52° 7′ N, 5° 17′34″ E:) was simulated in 3D using the Unreal Tournament 2004 game-engine v2.5 (Epic Games Inc.; see Figure 1 for the corresponding OpenStreetMap and Unreal aerial overviews). The area is enclosed by roads on four sides and contains blocks of houses, two squares with parking places, benches, and statues, two playgrounds with benches, and a network of pathways connecting the squares and playgrounds (for details see Figures 2–4). All houses have a garden in the back, typically enclosed with a wooden fence, with an exit door to a pathway. The pathways are typically covered with tarmac, and bordered on both sides with trees and shrubs. The houses are generally well maintained and quite uniform. The pathways and parks are reasonably well kept. The walking route (designated by arrows drawn on the ground) had no intersections and covered most of the area (Figure 1a). The route included three short segments of sidewalks along three different roads, several footpaths, two squares, and two playgrounds.

The VE represented a moderate socio-economic neighborhood. This type of environment was intentionally selected since there are indications that signs of disorder most effectively inspire fear of crime in higher socio-economic neighborhoods [118], [119]. A total of 42 test items were distributed over 34 different locations in the virtual environment. The items signaled three different classes of social incivilities: Neglect, Vandalism and Crime (see Table 1: [112], [114], [120]), and had social connotations ranging from indifference (e.g., litter, trash, dog droppings) and loitering (e.g. empty beer cans, cigarette butts, fast food wrappers) to vandalism (broken bus shelter windows) and predatory crime (smashed car windows, crime watch signs, CCTV cameras, and camera surveillance signs). Items that are typically associated with neglect, indifference, street youth and loitering, were collected at littered public sites and therefore had a realistic and weathered appearance. Broken car glass and old car tires were obtained from a local used car dealer. Rusted bicycle frames and wheels were obtained at a local junkyard. These experimental items were then either photographed (e.g., litter, warning signs) or modeled as 3D virtual objects (e.g., CCTV cameras, dog droppings) and placed in the virtual environment using the Unreal game editor.

**Table 1:** Experimental items, their connotations of physical and social disorder, and the experimental classification.

Experimental items (nr)	Social connotations	Experimental class (nr of items)
Garbage bags (2)	Neglect, indifference (Litter)	Neglect (24)
Cardboard boxes (1)		
Newspapers, flyers (2)		
Plastic shopping bags (2)		
Dog droppings (3)		
Bicycle frame (1)		
Bicycle wheels (2)		
Cigarette butts (1)		
Empty beer cans (7)		
Fast-food wrappers, boxes, paper cups (1)		
Old car tires (2)		
Bus shelter with broken windows (1)	Vandalism	Vandalism (1)
Smashed car windows and signs warning for car burglary (6)	Car burglary	Crime (17)
Neighborhood crime watch Signs (3)	Home burglary	
Signs that homes are protected by private security services (2)		
Signs that homes are protected by dogs (2)		
CCTV security cameras and signs (4)	Predatory crime	

Numbers in brackets indicate the number of items present in the VE.

The simulation was performed on Dell Precision 490 PC computers, equipped with Dell 19″ monitors. Logitech Rumblepad 2 Gamepads were used for navigation. User movement in the virtual environment was from a first-person viewing perspective with walking motion supporting forward and backwards movements and left and right rotation movements. User movement speed was fixed and collision detection enabled to prevent users from walking through objects. A non-repeating soundscape that was characteristic for the environment was composed from sounds (birds singing, cars passing by, children shouting, hammering and drilling, and dogs barking) recorded at several locations and at different times in the corresponding real environment. The soundscape was presented through Sennheiser eH 150 headphones. A previous study showed that this soundscape effectively increased the ecological validity of the VE while the absence of sound negatively biased the appraisal of the VE [4].

### Odor Selection

The scent of freshly cut grass was selected as the environmentally appropriate pleasant odor in this study. This scent is generally considered to be stimulating and refreshing. Since the VE used in this study shows a lot of vegetation, the scent of grass may evoke associations with well-kept property, and may also attract attention towards the greenery [86]–[88]. Hence, apart from its own stimulating effects, the scent of freshly cut grass may also indirectly improve mood and appraisal by focusing attention on the natural elements in the VE [121], [122]. In addition, a previous study found that the scent of freshly cut grass enhances the perceived quality of real-time animation of grass [123]. The smell of cut-grass was created by mixing ethanol with cis-3-hexenol (leaf alcohol) in a 9∶1 ratio. The associations that could be elicited by this scent were investigated by presenting it to a panel of 10 participants while they were viewing the VE. The scent was presented in small glass tubes containing a cotton swab with three to four drops of the solution and sniffed by the participants approximately 5 inches from their nose. Nine out of 10 participants reported associations with greenery (four mentioned grass, three named freshly cut leaves and one mentioned broken twigs).

An environmentally appropriate unpleasant scent was selected in a pilot test from a set of 8 candidate aversive smells. The candidate smells were respectively Burned Wood (RS/420), Reptile (RS/424), Diesel Fumes (RS/423), Metal (RS/426), Dusty (RS/425), Tar (RS/401), Cow Manure, and Natural Gas (all obtained from RetroScent, Rotterdam, The Netherlands: www.geurmachine.nl). The scents were identified by randomly assigned numbers, presented in small glass tubes containing a cotton swab with three to four drops of aroma oil, and sniffed by the 10 participants of the pilot test in random order, approximately 5 inches from their nose, while viewing the VE. The degree to which each scent fitted the VE (how environmentally appropriate the scent was for the VE) was evaluated on a 11 point semantic differential scale (ranging from 0 = *absolutely not* to 10 = *definitely*). Tar received the highest mean score (7.4), followed by Dusty (5.7). In addition, although the exact the nature of the tar smell was not identified by any of the testers, 8 out of 10 spontaneously reported associations with fire and burned material, while it was unanimously judged to be a very unpleasant scent that could occur in an environment as the one represented by the VE.

### Odor Diffusion

Scents were diffused in the room (about 25 m^2^) through a commercial electronic dispenser (1–3 RS-Classic Scentvertiser, RetroScent, Rotterdam, The Netherlands: www.geurmachine.nl). No odor was applied in the control condition. The dispenser was placed out of the participant’s sight behind a screen. The participants could not hear the sound of the dispenser when they wore their headphones and listened to the soundscape of the VE. The experimenter turned on the dispenser after the participants had started their tour through the VE and he turned it off before they were instructed to take off their headphones. Odor was intermittently diffused (with a cycle period of 1 minute) during the experiment so that the participants received fluctuating concentrations over time, thus preventing full adaptation. The perceived odor intensity should neither be overwhelming (to avoid eliciting inappropriate expectations in the participants: [124]) nor too low (so that the odor stimulation would be ineffective). Ideally, it should be sufficiently strong to be just noticeable when attended to. Previous studies found that the influence of odors on visual stimuli is largest when the odors are presented at near detectable levels [61], [68], [125]. The odor intensity used in this study was between low and intermediate, corresponding to a mean level between 3 and 5 on a 10-point scale. A pilot experiment was performed to determine a setting of the dispenser and a duty cycle that resulted in a mean rating of 5. The room in which the test was performed was well ventilated prior to each session. Only one scent per day was diffused to avoid mixing odors, and the lab was fully ventilated overnight to remove any lingering trace of the scent. Before beginning the study each morning, the room was ‘sniff-tested’ by the two experimenters; no odors were detected to have remained in the room.

### Instruments

#### General questionnaire

As the results may be influenced by the characteristics of the participants, they were asked to complete a *General Questionnaire* including socio-demographic measures (sex, age, and education). Education was clustered into four groups: middle and higher level education, academic education, and other types of education.

#### Game and computer experience questionnaire

A 6-item *Game and Computer Experience Questionnaire* (Table 2; see also [50]) was designed to assess the participants’ general experience with computers, games and virtual environments. Three items (nrs. 1,2 and 6) are related to the total amount of time spent on playing computer games (each was scored on a scale from 0 = no time spent to 3 = a large amount of time spent), two items (nrs. 4 and 5) address the experience with navigating virtual environments (one item is scored as 0 = no experience and 1 = some experience, the other item merely serves to check what type of environment was navigated), and the kind of games played (item nr. 3). The total score on the *Game and Computer Experience Questionnaire* ranges from 0 (no time spent on games and no experience with virtual environments) to 18 (a large amount of time spent on many different games and experience with virtual environments). Scores ranging from 0 to 5 correspond to low experience, scores from 6 to 12 indicate average experience, while scores from 13 to 18 correspond to extensive experience.

**Table 2:** *Game and Computer Experience Questionnaire*, together with the scores attributed to each item.

Nr	Item	Answer	Score
1	How frequently do you use a computer?	Never	0
		A few times a month	1
		A few times a week	2
		Daily	3
2	How many hours per week do you spend playing games(e.g. on a PC, Playstation, Xbox, Nintendo etc.)?	0 hours (*please proceed to Question 4*)	0
		1 to 5 hours	1
		5 to 10 hours	2
		More than 10 hours	3
3	What kind of games do you play *(you can choose more than one type)*	Shooting games	1
		Fighting games	1
		Sport games (e.g. racing, football, etc.)	1
		Adventure games (e.g. Mario, Zelda, etc.)	1
		Simulation games (e.g. SIMS, etc.)	1
		Role playing games	1
		Strategy games	1
		Other, i.e. …………………….	1
4	Did you ever explore a virtual environment?	Yes	0
		No (*if not the questionnaire ends here*)	1
5	What kind of virtual environment did you explore (e.g. Second Life,Active World, a simulation, a training)?	…………………….	
6	How many hours per week do you spend using a virtual environment?	0 hours	0
		1 to 5 hours	1
		5 to 10 hours	2
		More than 10 hours	3

#### Fear of crime questionnaire

A 7-item *Fear of Crime Questionnaire* (see Table 3) was applied to measure the participants’ affective appraisal of a given situation, i.e., their interpretation of cues in the environment that signal potential threats and that may evoke a chain of thoughts about unpleasant events (equivalent to Jackson’s *Worry about Crime* concept: [15]; see also [22]). This questionnaire contained six statements with connotative meanings related to concern for personal safety. Five items (nrs. 1–5) were adapted from a validated *Perceived Danger* questionnaire [126]. One statement (item 6) was added as a check (for item 2), and one statement (item 7, from [118]) was added to measure concern for personal property. The items were scored on a 5-point Likert scale (ranging from 1 = *completely disagree* to 5 = *completely agree*).

**Table 3:** Factor loadings of *the Fear of Crime Questionnaire* items.

		Factors	
Nr.	Fear of Crime Items	Concern forPersonal Safety	Concern for PersonalProperty
1	I would go a long way around to avoid this place.	.85	.21
2	I feel/would feel uneasy at this place.	.85	.45
3	I would make haste to get away from this place.	.80	.37
4	I would not mind to walk alone here at night-time.	.78	.29
5	I have/would have an unpleasant feeling in this place.	.76	.45
6	I feel/would feel safe at this place.	.69	.49
7	I would leave my bicycle at this place without concern.	.29	.94

The two main factors are Concern for Personal Safety and Concern for Personal Property.

#### Mental state questionnaire

A 7-item *Mental State Questionnaire* (adapted from [127]), consisting of four negative (*agitated, angry, anxious, distressed*), two neutral (*calm, relaxed*), and one positive (*cheerful*) affective terms served to assess the affective connotations elicited by the individual incivilities. For each individual sign of disorder which they encountered during their walk participants reported their affective connotation by selecting one of the 7 items (“*I feel/would feel … at this place.*”).

#### Post-experiment questionnaire

A 4-item *Post-Experiment Questionnaire* contained three questions investigating the extent to which the ambient temperature, illumination and atmosphere in the room were characteristic for the VE (these three items were scored on a 5-point Likert scale, ranging from 1 = *completely disagree* to 5 = *completely agree*) and an open question (“*Was there anything else you noticed during the experiment*?”) to test if the participants had noticed the ambient scent in the room.

### Experimental Procedure

After their arrival at the laboratory, the participants first read and signed an informed consent form. Next, they filled out both the *General Questionnaire* and G*ame Experience Questionnaire*. Then they read the following instructions:

“*The experiment concerns an area of Soesterberg near the TNO lab, and will take about 45 minutes. Citizens living in this area are concerned about the increasing social disorder in their neighborhood. They intend to draft a plan of action to confront this problem. After making an inventory of the different types of incivilities occurring in their neighborhood, the citizens will prioritize the order in which these should be addressed. To enable a large number of people to give their opinion on the social disorder in this area, the concerned citizens have commissioned a realistic and highly detailed computer model of their neighborhood.*

*It is your task to make a tour through this virtual model and assess the social disorder in this neighborhood. Your route is marked by arrows drawn on the ground. Each time you notice signs of incivilities (e.g., litter, dog droppings, broken car windows, etc.) during your inspection tour, you are requested to:*

*1. Make a snapshot of each sign of incivilities you notice (by pressing key F12).*

*2. Enter a brief description of the incivility on your questionnaire.*

*3. Rate any concerns for your personal safety which you associate with the perceived incivility using the ‘Fear of Crime Questionnaire’.*

4. *Describe any affective feelings you associate with the perceived incivility by choosing one of the 7 affective terms on the ‘Mental State Questionnaire’ (agitated, angry, anxious, distressed, calm, relaxed, cheerful)*”.

Next, the experimenter verified if the participants had understood their instructions, and started the simulation. The experimenter then explained the function of the gamepad, and gave the participant the opportunity to practice maneuvering through the virtual environment for about 5 minutes. At the end of this practice period the experimenter checked if the participant was able to perform the required maneuvers, and whether the participant paid attention to the arrows on the ground and the signs of disorder. Then, the experimenter gave the participants the printed questionnaires which they could use to fill out their reports, and positioned the point-of-view in the virtual environment at the starting location, facing the direction of the route. The participants then put on their headphones and started their walkthrough, which they performed at their own pace. Each time the participants noticed signs of disorder during their walk they reported the item they had noticed and filled out the *Fear of Crime Questionnaire* and the *Mental State Questionnaire*. During the test, the experimenter was seated behind a screen in the room and intermittently turned on the odor dispenser at one minute intervals, maintaining a slightly fluctuating near threshold ambient odor level. Finally, after finishing their walkthrough, the participants filled out the *Post-Experiment Questionnaire*.

The experimental protocol was reviewed and approved by the TNO internal review board on experiments with human participants (TNO Toetsings Commissie Proefpersoon Experimenten, Soesterberg, The Netherlands), and was in accordance with the Helsinki Declaration of 1975, as revised in 2000 [128]. The participants provided their written informed consent prior to testing. The participants received a modest financial compensation for their participation.

### Pre-trial

Prior to the main experiment, a pilot test including 4 participants was performed to test the entire experimental procedure. It was confirmed that the instructions were clear, that the participants could easily find their way through the environment, that the signs of disorder were salient enough to be noticed, and that the setting of the electronic dispenser (near threshold, as subjectively determined by the experimenters) indeed resulted in a just noticeable level of ambient door.

### Participants

The main experiment was performed by 70 participants (40 males and 30 females) that were selected from the TNO database of volunteers, with an average age of 43 years (M = 43, SD = 17). The selection criteria guaranteed that they were not familiar with the urban area represented by the VE, that they had no problems with their sense of smell, and that they all had normal (or corrected to normal) vision with no color deficiencies. Also, they were unaware of the aim of the experiment. All participants were educated: 23% had received academic education, 44% had received higher education, 23% had received middle level vocational education, and 10% had received some other form of education. All participants had computer experience: 34% of the total population played computer games on regularly base, while the remaining 66% had at least some occasional experience with VE’s. The participants’ mean age, level of education, and computer proficiency and game experience were approximately the same for all three (no-ambient smell, ambient tar odor, and ambient grass odor) experimental conditions (see Table 4).

**Table 4:** Distribution of participants over the experimental conditions.

	Condition					
	Control		Tar odor		Grass odor	
	male	female	male	female	male	female
Sex (nr)	14	7	14	13	12	10
Average age (years)	42.71		43.26		41.73	
Computer and game experience (average level)	9		9		10	
Middle level education (%)	24		30		14	
Higher level education (%)	43		30		64	
Academic level education (%)	19		33		14	
Other type of education (%)	14		7		9	

### Data Analysis

The participant-variable ratio did not allow for a straightforward statistical analysis of the combination of 7 fear of crime scores for 42 items of social incivilities in 3 conditions. Therefore a data reduction strategy was applied. Factor analysis was used to cluster the *Fear of Crime* statements in the two scales *Concern for Personal Safety* and *Concern for Personal Property*. The internal consistency of the resulting scales was checked by computing Cronbach’s alphas. Next, scale scores were calculated based on the average of the statements’ scores within a scale, such that higher scores represented more fear of crime. The relation between the two scales was calculated using Pearson’s correlation. In addition, the 42 experimental items were clustered in three classes based on their connotations: Neglect, Vandalism and Crime (see Table 1). Analysis of variance was used to test the relationships between the main variables. The statistical analyses were performed with SPSS 20.0 for Windows (SPSS, Chicago, Ill., USA). For all analyses a probability level of p<0.05 was considered to be statistically significant.

## Results

The *General Questionnaire* provided information about the age, sex and education of the participants, while the *Game and Computer Experience Questionnaire* served to assess their experience with computers, games and VEs. Table 4 shows how the participants in this study were distributed over the experimental conditions with respect to these factors. The different experimental groups were similar in average age and game and computer experience, but differed slightly in sex and education. Since multivariate analysis showed no main or interaction effects of age, level of education, and computer experience on fear of crime, these factors were omitted from later analyses.

The *Fear of Crime Questionnaire* served to measure whether signals of social disorder evoked safety related concerns. Table 3 shows the results of a factor analysis of the 7 statements from the *Fear of Crime Questionnaire*. Six variables load onto a single factor, which accounts for 84% of the total variance and has a high internal consistency (Cronbach’s alpha = 0.93). The six items which load onto this factor are all related to *Concern for Personal Safety* (physical harm, violence). The remaining item (“*I would leave my bicycle at this place without concern*.”) is related to *Concern for Personal Property* (theft, vandalism) and accounts for 7% of the total variance.

Next, we calculated the average of the statements’ scores within both scales, such that higher scores represented more fear of crime (see Tables 5 and 6). Both factors are significantly correlated (r = .63, df = 1565, p<.00): *Concern for Personal Property* increases when *Concern for Personal Safety* increases.

**Table 5:** Mean (SD) of the factor *Concern for Personal Safety* in the control, ambient tar and ambient grass odor conditions for each of the three classes of experimental items signaling respectively Neglect (24 items), Vandalism (1 item) and Crime (17 items: see Table 1).

	Signals of		
Condition	Neglect	Vandalism	Crime
Control	2.90 (.64)	3.22 (.85)	3.02 (.82)
Tar odor	2.62 (.72)	2.67 (.84)	3.02 (.82)
Grass odor	2.64 (.68)	3.04 (1.20)	2.91 (.87)

**Table 6:** Mean (SD) of the factor *Concern for Personal Property* in the control, ambient tar and ambient grass odor conditions for each of the three classes of experimental items signaling respectively Neglect (24 items), Vandalism (1 item) and Crime (17 items: see Table 1).

	Signals of		
Condition	Neglect	Vandalism	Crime
Control	3.66 (.91)	4.05 (1.08)	3.20 (.88)
Tar odor	3.42 (.68)	3.94 (.80)	3.38 (.81)
Grass odor	3.52 (.83)	3.99 (1.20)	3.43 (.80)

Then, the affective connotations reported for the detected signs of disorder (from the combination of the *Mental State* and *Fear of Crime Questionnaires*) were clustered for each of the three classes of experimental items: Neglect, Vandalism and Crime (see Table 1). There is a significant difference (χ^2^ = 18.94; df = 4; p = <.05) between the observed and expected frequencies of the affective connotations (negative, neutral, or positive) associated with the reported items (signs of incivilities) in the classes Neglect, Vandalism and Crime. Items in the classes Vandalism and Crime were more frequently associated with negative affective connotations than items in the class Neglect. However, there is no difference in *Concern for Personal Safety* associated with reports on experimental items in different experimental classes. Also, items that could inspire *Concern for Personal Property* (e.g., signs of home and car burglary, abandoned bikes) did not elicit any crime related concerns.

The hypotheses H1 and H3 - that participants in the (un)pleasant odor condition would report more (less) concern for crime than participants in the control condition - are both not proven by this study: analysis of variance showed no significant difference in the factors *Concern for Personal Safety* and *Concern for Personal Property* between the control condition and each of the two odor conditions (Table 7), for each of the experimental classes. Also, the hypotheses H2 and H4 - that participants in the (un)pleasant odor condition would associate more (less) negative affect with these signs because they would (unconsciously) attribute the affective quality of the ambient odor to the VE – are both not supported by the present results: the affective connotations associated with the reported items do not differ between the control and each of the two experimental (ambient odor) conditions.

**Table 7:** Results of a two-way ANOVA to test the difference between the factors *Concern for Personal Safety* and *Concern for Personal Property* in respectively the control and the two ambient odor conditions.

Factor	Control - Tar	Control - Grass
*Concern for* *Personal Safety*	F_1,42_ = .02; p = .88	F_1,37_ = .41; p = .53
*Concern for* *Personal Property*	F_1,42_ = 1.21; p = .28	F_1,37_ = .59; p = .45

Overall, women were significantly more concerned about their own personal safety than men (F_1,61_ = 14,93, p<.00).

In response to the open question in the *Post-Experiment Questionnaire* only one participant (out of 23) claimed to have noticed a (Lysol) smell in the room in the control condition. In the tar odor condition one participant (out of 23) reported to have noticed a smell, but he was unable to identify its nature, and did not link the odor to the experiment. No participant noticed a smell in the grass odor condition. Five participants (out of 70) reported that they experienced the absence of people in the VE as frightening.

## Discussion and Conclusions

There was no significant difference in the factors *Concern for Personal Safety* and *Concern for Personal Property* between the control condition and each of the two odor conditions. Also, the affective connotations associated with the reported items do not differ between the experimental conditions. The present results therefore appear to falsify the hypotheses that the hedonic quality (pleasant or unpleasant) of an environmentally congruent ambient odor would modulate the affective evaluation of the desktop VE (H1 and H3) and the affective connotations the participants attribute to signs of disorder therein (H2 and H4). However, they agree with earlier reports that ambient scent has no effect on shopping behavior [129], [130]. In particular, Schifferstein and Blok [130] found that the scent of freshly cut grass did not affect sales of thematically (in-) congruent products. These authors argue that ambient scent is probably more diagnostic for the physical environment of the observer than for the particular items in that environment. This suggests that a close spatiotemporal link between the visual cues in a desktop VE and the scents with which they are supposed to be associated may be required to effectively establish these associations. Hence, it would be interesting to investigate more immersive VEs that can convincingly induce the illusion that scents emanate from the objects that are displayed in the scene.

Experimental items signaling vandalism (e.g., a damaged bus shelter) and crime (e.g., home protections signs and cameras) more frequently evoked negative affective appraisals than items representing neglect (e.g., litter, dog droppings, old bicycle parts). This finding agrees with the discriminant validity of different types of perceptual incivilities that is also found in the real world (e.g., between crime and social incivilities: [131], [132]). In reality, signs of crime are also more likely to evoke negative appraisals since they are typically associated with the risk of personal victimization [133].

In response to the open question in the *Post-Experiment Questionnaire* six participants stated that they had experienced the absence of people (avatars) in the simulation as discomforting. This agrees with previous real world observations that lack of social presence evokes fear of victimization and determines navigation behavior, especially in women [134], [135].The current finding that women were significantly more concerned about their own personal safety than men also agrees with real world observations [134], [135].

The fact that only one participant noticed a smell in an odor condition (but failed to identify it or link it to the experiment) suggests that the ambient odors were indeed successfully presented at a just noticeable intensity level.

Summarizing, although semantically congruent ambient scents of different hedonic quality failed to influence the affective appraisal of the desktop VE, the finding that signs of crime were more frequently associated with negative feelings and the fact that women were more concerned about their personal safety than men suggest that the affective appraisal of the VE had at least some ecological validity.

### Limitations of this Study

The results show that visual cues in the VE signaling vandalism and crime indeed evoked negative affective appraisals, just like they do in reality [48], [114]. However, except for broken car glass, the experimental items used in this study represent minor offenses and have no obvious connotations of violence or predatory crimes that may inspire concern of personal victimization. Indicators of serious (prostitution, drug selling, harassment) or even less serious (public drunkenness) social disorder may elicit crime related concerns more effectively, but these are typically not present in daylight hours [136] and are not easily implemented in experimental conditions.

The (physical and demographic) characteristics of lower socio-economic neighborhoods can mute the effects of disorder (e.g. [112], [137], [138]). A VE representing a higher socio-economic neighborhood was therefore selected, in the expectation that this type of environment would be more susceptible to the effects of disorder, resulting in larger differences between fear of crime levels in respectively the pleasant and unpleasant odor conditions ([118]). However, the aesthetic appeal of the simulated neighborhood used in this study may also have muted the effects of the signs of disorder.

As noted before, the desktop VE and task used in this study were not designed to induce a strong sense of immersion or presence. As a result, the participants may have unconsciously associated the ambient scent with their physical environment and not so much with the VE they explored. Schifferstein and Blok [130] also argued that ambient scent is probably more diagnostic for the physical environment of an observer than for the particular items in that environment.

### Suggestions for Future Research

The present study investigated the effects of ambient scent on the affective appraisal of a desktop VE with static contents. In future experiments the realism of the simulation can be enhanced by adding dynamic visual, auditory, olfactory and haptic features that interact with the user. Dynamic audio cues could include voices that shout or dogs that bark when participants walk by. Dynamic visual cues could be passing traffic (cars, motorbikes), virtual humans that drop litter, create graffiti, shout insults or make obscene gestures when approached, or dogs that growl at people passing by. In everyday life ambient smells are important and essential environmental cues that trigger a wide range of different emotions and associations. Dynamic olfactory cues could include location dependent smells like urine, sewage or garbage smells in alleys, or gasoline smells near cars [93]. Finally, dynamic haptic cues could include sudden gusts of air when moving around a corner or constant breezes when walking through open spaces [139]. Each of these modifications may serve to make the VE more effectively elicit cognitive and affective responses similar to those that users would have to corresponding real environments [140], [141].

Participants in this study were not familiar with the urban area represented by the VE. Since it is known that scenes more effectively elicit affective feelings when they have personal relevance [142], it would be interesting to test whether ambient odors can affect the appraisal of scenes with high personal relevance. Personal relevance can for instance be achieved through background stories suggesting that the VE experience will have personal implications (e.g., a suggested follow-up task like a nighttime visit to the VE’s real world counterpart).

The present study tested only two different scents. A wider range of scents with various hedonic qualities should be investigated to assess whether olfactory cues can bias the affective appraisal of a desktop VE. In combination with gaze tracking, the use of multiple scents that are semantically related to the contents of a VE may help to assess whether olfactory cues can bias visual attention.

In simulation studies social incivilities inspire safety related concerns far more effectively than physical incivilities [3], just like in reality [113]. In the current simulation social presence was merely implied through the occasional sound of voices, traffic, birds, music and hammering in the ambient sound track. There was no visual evidence of human or animal activity. The soundtrack played independently of the actions of the user and had no visual counterpart. It is known that ambient odor can significantly affect the way people interact in real life: people display more social behavior [57], [60], [143], [144] and find each other more attractive [145] in the presence of pleasant ambient odors, whereas they become more frustrated [70] and find each other less attractive [146], [147] in the presence of malodorants. Future studies should investigate whether these real-life effects of ambient odors also transfer to the interaction between users and avatars in a VE.

## Data Availability

The experimental data and additional information on this study are deposited in the Dryad Repository: http://dx.doi.org/10.5061/dryad.f37km.
